# Supplementary material for: Unified theoretical framework for black carbon mixing state allows greater accuracy of climate effect estimation
Source: Nat Commun. 2023 May 10;14:2703. doi: 10.1038/s41467-023-38330-x (PMC10172310; doi:10.1038/s41467-023-38330-x)
Supplement: Supplementary file 1 — Supplementary Information [file 41467_2023_38330_MOESM1_ESM.pdf]

Supplementary Information for Unified theoretical framework for black carbon mixing state allows greater accuracy of climate effect estimation

Authors: Jiandong Wang[1,2*†], Jiaping Wang[3,4*†], Runlong Cai[5], Chao Liu[1,2], Jingkun Jiang[6], Wei Nie[3,4], Jinbo Wang[3], Nobuhiro Moteki[7], Rahul A. Zaveri[8], Xin Huang[3], Nan Ma[9], Ganzhen Chen[2], Zilin Wang[3], Yuzhi Jin[2], Jing Cai[5], Yuxuan Zhang[3,4], Xuguang Chi[3,4], Bruna A. Holanda[10,13], Jia Xing[6], Tengyu Liu[3,4], Ximeng Qi[3,4], Qiaoqiao Wang[9], Christopher Pöhlker[10], Hang Su[10], Yafang Cheng[10], Shuxiao Wang[6], Jiming Hao[6], Meinrat O. Andreae[10, 11, 12], Aijun Ding[3,4*]

Affiliations:

[1]Collaborative Innovation Center on Forecast and Evaluation of Meteorological Disasters, Nanjing University of Information Science and Technology, Nanjing 210044, China;

[2]China Meteorological Administration Aerosol-Cloud-Precipitation Key Laboratory, School of Atmospheric Physics, Nanjing University of Information Science and Technology, Nanjing 210044, China;

[3]Joint International Research Laboratory of Atmospheric and Earth System Sciences, School of Atmospheric Sciences, Nanjing University, Nanjing, 210023, China;

[4]National Observation and Research Station for Atmospheric Processes and Environmental Change in Yangtze River Delta, Nanjing, 210023, China

[5]Institute for Atmospheric and Earth System Research / Physics, Faculty of Science, University of Helsinki, Helsinki, 00014, Finland;

[6]State Key Joint Laboratory of Environment Simulation and Pollution Control, School of Environment, Tsinghua University, Beijing 100084, China;

[7]Department of Earth and Planetary Science, Graduate School of Science, The University of Tokyo, Tokyo, 113-0033, Japan;

[8]Atmospheric Sciences & Global Change Division, Pacific Northwest National Laboratory, Richland, Washington 99352, United States;

[9]Institute for Environmental and Climate Research, Jinan University, 511443, Guangzhou, China.

[10]Max Planck Institute for Chemistry, 55128 Mainz, Germany;

[11]Scripps Institution of Oceanography, University of California San Diego, La Jolla, CA 92093, USA;

[12]Department of Geology and Geophysics, King Saud University, 11451 Riyadh, Saudi Arabia.

[13]Now at: Hessian Agency for Nature Conservation, Environment and Geology, 65203, Wiesbaden, Germany

[†]These authors contributed equally to this work.

[*]Corresponding author. Email: jiandong.wang@nuist.edu.cn; wangjp@nju.edu.cn; dingaj@nju.edu.cn

Theoretical derivation

The theoretical derivation in the main text is based on a Lagrangian coordinate system in D_p-space, which is more intuitive. Here the D_p-space means the space where D_p is the scale of distance and particle sizes can be represented as points. The growth of particles can be considered as the movement in D_p space. In Lagrangian coordinates, the change in size of each individual particle is tracked. A more rigorous expression can also be performed using a Eulerian coordinate system in D_p space (in which the number concentration of aerosols in a specific D_p bin is tracked). The general dynamic equation for aerosol number concentration $n(= dN / dD_p)$ in the accumulation mode is,

$$\frac{\partial n(D_p)}{\partial t} + R_{emis} + GR(D_p)\cdot\frac{\partial}{\partial D_p}n(D_p) + Dep(D_p)\cdot n(D_p) = 0 \quad \text{(S1)}$$

where the second, third and fourth terms represent the impact of emission, growth, and deposition processes. The growth rate of bins in the accumulation mode is related to the particle diameter, thus $GR(D_p)$ is used. Further, the deposition rate is also related to the particle diameter and $Dep(D_p)$ is used to represent the deposition rate of D_p.

For size bins without emission, $R_{emis} = 0$, and Eq. (S1) could be simplified as

$$\frac{\partial n(D_p)}{\partial t} + GR(D_p)\cdot\frac{\partial}{\partial D_p}n(D_p) + Dep(D_p)\cdot n(D_p) = 0 \quad \text{(S2)}$$

Based on the steady-state approximation, $n(D_p)$ does not vary much with time. Thus,

$$\frac{\partial n(D_p)}{\partial t} = 0 \quad \text{(S3)}$$

Combining Eq. (S2) and Eq. (S3), we obtain,

$$GR(D_p)\cdot\frac{\partial}{\partial D_p}(n(D_p)) = -Dep(D_p)\cdot n(D_p) \quad \text{(S4)}$$

Therefore,

$$\frac{-Dep(D_p)}{GR(D_p)} = \frac{\frac{\partial}{\partial D_p}n(D_p)}{n(D_p)} = \frac{\partial}{\partial D_p}ln(n(D_p)) \quad \text{(S5)}$$

To get the analytical equation, we assume Dep and GR follow a power-law dependence of D_p, $Dep \sim D_p^{a}$, $GR \sim D_p^{b}$,

$$ln(n(D_p)) = ln(n(D_c)) - \frac{Dep}{(a-b+1)\cdot GR}\cdot(D_p^{(a-b+1)} - D_c^{(a-b+1)}) \quad \text{(S6)}$$

The D_p distribution with different relationships between a and b is shown in Fig. S1. The derivation in the main text (Eq. 5) can be considered as a special case when a=0 and b=0. Our field observation results (Fig. 2) confirm that ln($n(D_p)$) and D_c are in a linear relationship and indicate that $a \sim b$ in Eq. S6. Eq. 5 may look similar to those derived for total aerosols in the pioneering works by Junge[33] and Willeke & Whitby[34], but in fact they are different in both equation format and physical mechanism. We find that the size distributions of BC-containing particles follow an exponential law, while they derived a power law and multimodal distribution

for total particle. This difference can be also seen if we present our results with different x axes (see Fig. S2).

In addition to the dependency of *GR* and *Dep* on D_p, the variations of *GR* and *Dep* as a function of time should also be considered. The lifetime of BC aerosols is 3-10 days. Therefore, long-term variations of *GR* and *Dep*, such as seasonal variations, do not affect the assumption of steady-state, and our theoretical framework is applicable. The suitability of this theory under the influence of short-term periodical variations of *GR* and *Dep* (most importantly the diurnal variation) is discussed here.

GR and *Dep* are assumed to have a periodical variation with cycling time of τ, that is,

$$\int_t^{t+\tau} GR(t)dt = \overline{GR} \cdot \tau \tag{S7}$$

$$\int_t^{t+\tau} Dep(t)dt = \overline{Dep} \cdot \tau \tag{S8}$$

Hence, Eq. 2 and Eq. 4 can be represented as Eq. S9 and Eq. S10, respectively.

$$\begin{aligned} \Delta D_p &= \int_t^{t+\tau} GR(t)dt \\ &= \overline{GR} \cdot \tau \end{aligned} \tag{S9}$$

$$\begin{aligned} ln(\frac{n(t+\tau)}{n(t)}) &= \int_t^{t+\tau} -Dep(t)dt \\ &= -\overline{Dep} \cdot \tau \end{aligned} \tag{S10}$$

It can be observed that Eq. S9 and Eq. S10 have similar formats as Eq. 2 and Eq. 4, only with $\overline{GR}$ and $\overline{Dep}$ instead of *GR* and *Dep*, and τ instead of *t*.

Therefore, the assumption of constant *GR* and *Dep* (independent of time) is applicable to periods that are integer multiples of τ or periods much longer than τ. This assumption cannot be used to describe BC mixing states during some fast and non-periodic meteorological condition changes (e.g., a passage of cold front and precipitation). When discussing the mixing state of BC, multiday statistics are often adopted to represent its average condition, in which case the above derivation can be used. Moreover, the steady-state assumption is also applicable for us to determine the overall mixing state of BC on a large scale, which is one of our major targets in this study.

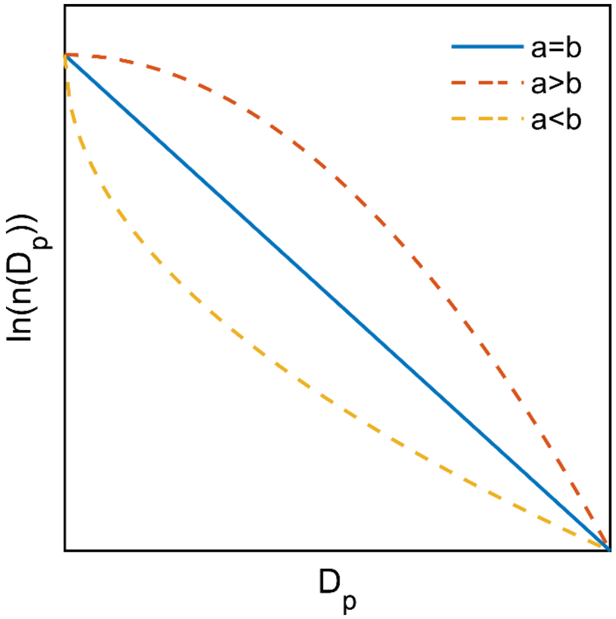

Fig. S1.

Theoretical relationship between *ln(n(D_p))* and the diameter of BC-containing particles (D_p) under three conditions. The blue line represents the case demonstrated in the main text.

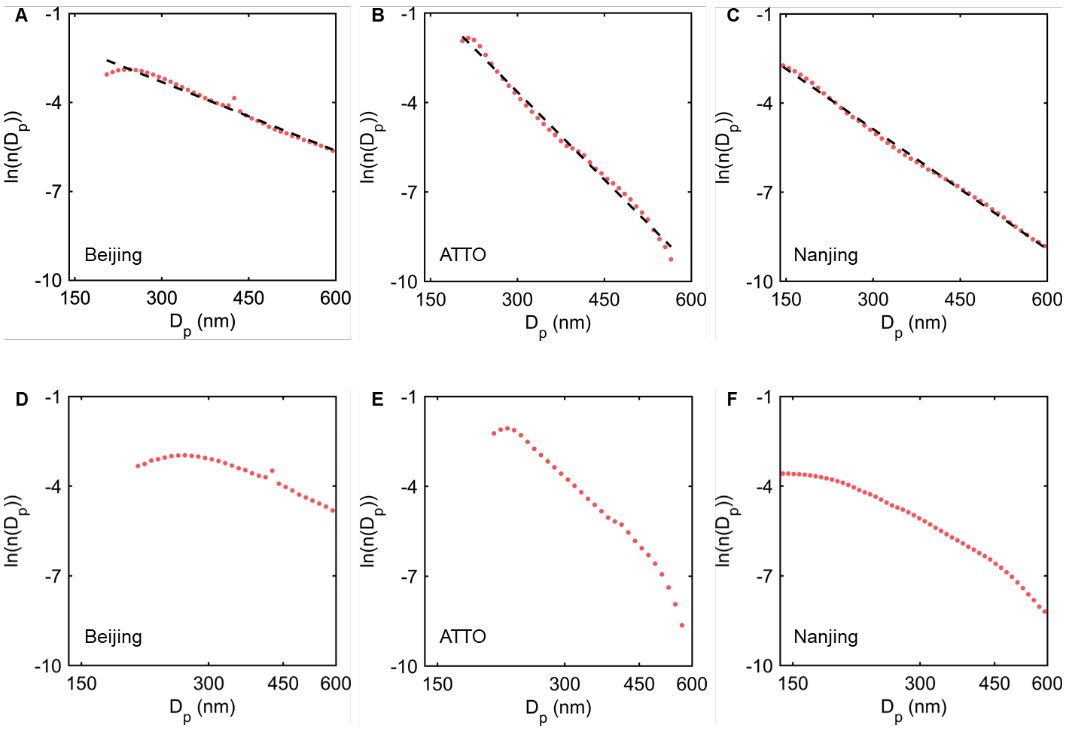

Fig. S2.

Black carbon (BC) size distributions using different coordinates from field measurements. (A-C) BC size distribution using a logarithmic scale on the y-axis and linear scale on the x-axis; (D-F) BC size distribution using a logarithmic scale on both x -axis and y-axis.

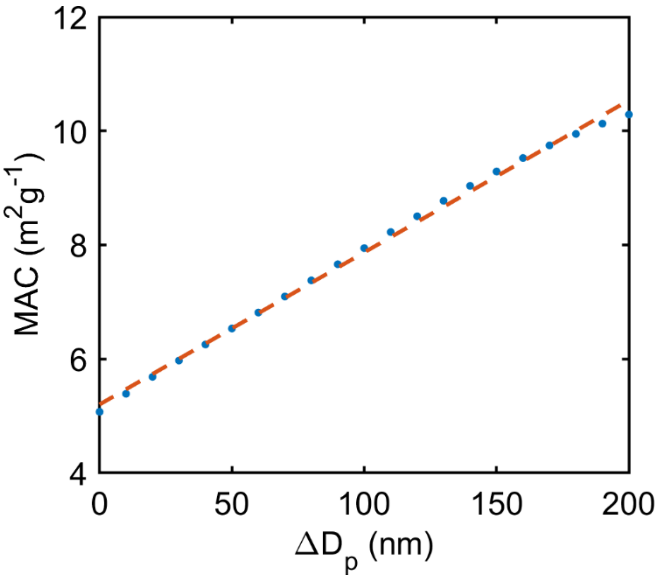

Fig. S3.

Change of mass absorption cross-section (MAC) of black carbon (BC) with coating thickness (ΔD_p). Blue dots represent the calculated MAC based on core-shell Mie theory with the linear fit shown as the red line.

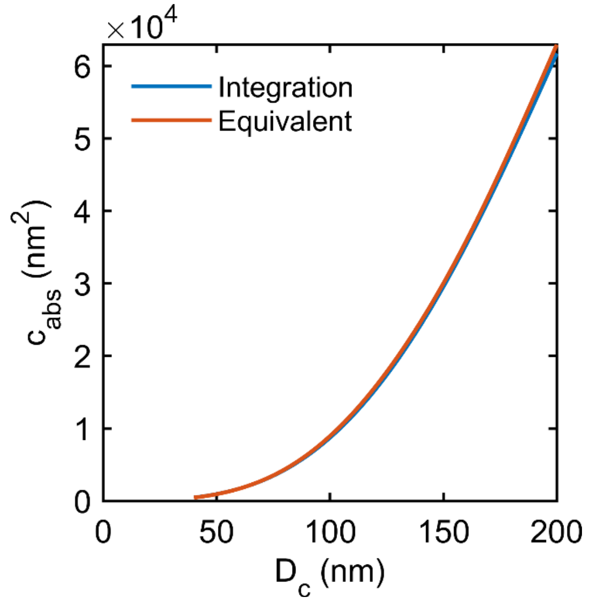

Fig. S4.

Black carbon (BC) absorption coefficient (*C_{abs}*) of different BC core size (*D_c*) calculated using equivalent diameter of BC-containing particles (equivalent *D_p*) and *D_p* distributions. The red line represents the calculated c_{abs} using the equivalent D_p. The blue line stands for the calculated c_{abs} using the integrated D_p from the D_p distribution.

Table S1.

Observation periods and site types for the single particle soot photometers (SP2) measurements

Site	Observation period	Type	Reference
Nanjing, China	1/2/2020–28/2/2020<br>1/4/2020–30/4/2020<br>1/12/2021–31/12/2021	Suburban	This study
Lulang, China	1/4/2021–25/5/2021	Background in the Tibetan Plateau	This study
Maqu, China	26/6/2021–8/7/2021	Rural in the Tibetan Plateau	This study
Shaoguan, China	4/12/2020–10/12/2020	Rural	This study
Beijing, China	13/11/2014–3/12/2014	Urban	Zhang et al., 2018 [3]
Sacramento, USA	14/6/2010–15/6/2010	Urban with biomass burning influence	Zaveri et al., 2012 [4]
Tokyo, Japan	2/8/2012–8/8/2012	Urban	Moteki et al, 2014 [5]
Amazon Tall Tower Observatory (ATTO), Brazil	23/10/2019–31/10/2019	Biomass burning	

Table S2.

The ratio between the aerosol absorption coefficients before and after removal of coating (E_{abs}) of black carbon (BC) from field observations.

Longitude Latitude	Location	Wavelength (nm)	Sampling duration	E_{abs}	References
43.66°N 79.39°W	Toronto Canada	760	2006.12-2007.1	1.21 [1.02-1.43]#	Knox et al., 2009 [6]
38.64°N 121.35°W	Sacramento, USA	405	2010.6.17-2010.6.29	1.13±0.01	Cappa et al., 2012 [7]
		532		1.06±0.006	
40.02°N 105.27°W	Boulder USA	404	2010.9	1.5	Lack et al., 2012 [8]
		532		1.3	
51.05°N 0.12°W	London UK	405	2012.2	1.3	Liu et al., 2015 [9]
		781		1.4	
37.50°N 137.40°E	Noto Peninsula, Japan	405	2013.4.17-2013.5.14	0.99 [0.87-1.06]*	Ueda et al., 2016 [10]
		532		1.06 [0.93-1.20]*	
		781		1.23 [1.10-1.35]*	
32.06°N 118.70°E	Nanjing, China.	405	2014.8.16-2014.8.28	1.41±0.39	Ma et al., 2020 [11]
		532		1.42±0.40	
		781		1.35±0.38	
36.81°N 119.78°W	Fresno, USA	405	2014.12.25-2015.1.12	1.37±0.22	Cappa et al., 2019 [12]
		532		1.22±0.15	
		870		1.10±0.13	
34.10°N 117.49°W	Fontana USA	405	2015.7.3-2015.7.15	1.10±0.27	Cappa et al., 2019 [12]
		532		1.07±0.22	

Measured E_{abs} using thermodenuder (TD) method was listed in above table. TD method removes coating material by heating the sample in a TD, then defined E_{abs} with $\mathrm{E_{abs}} = b_{abs;ambient}/b_{abs;TD}$, where $b_{\mathrm{abs;TD}}$ is corrected for particle losses.

#E_{abs} in Knox et al., 2009 [4] was obtained from $MAC_{unheated}/MAC_{heated}$. The range represents the E_{abs} of aerosol with different age category.

*E_{abs} range was the 25th-75th percentile.

Table S3.

Densities and refractive indices for shortwave radiation of the species in the CESM-CAM6 and WRF-Chem simulations.

Species	Density (g cm^{-3}) (CESM-CAM6)	Density (g cm^{-3}) (WRF-Chem)	Refractive index (CESM-CAM6)	Refractive index (WRF-Chem)
Black carbon	1.7	1.8	1.95 + 0.79i	1.85 + 0.71i
Organic matter	1.0	1.0	1.53 + 0.0057i	1.45 + 0i
Dust	2.6	2.6	1.56 + 0.0019i	1.55 + 0.003i
Sulfate	1.77	1.8	1.43 + 0i	1.45 + 0i
Nitrate	1.77	1.8	1.5 + 0i	1.45 + 0i
Ammonia	1.77	1.8	1.5 + 0i	1.45 + 0i

Supplementary References

1. Junge, C. E. The size distribution and aging of natural aerosols as determined from electrical and optical data on the atmosphere. *Journal of Meteorology* **12**, 13–25 (1955).
2. Willeke, K. & Whitby, K. T. Atmospheric Aerosols: Size Distribution Interpretation. *Journal of the Air Pollution Control Association* **25**, 529–534 (1975).
3. Zhang, Y. *et al.* Amplification of light absorption of black carbon associated with air pollution. *Atmos. Chem. Phys.* **18**, 9879–9896 (2018).
4. Zaveri, R. A. *et al.* Overview of the 2010 Carbonaceous Aerosols and Radiative Effects Study (CARES). *Atmos. Chem. Phys.* **12**, 7647–7687 (2012).
5. Moteki, N., Kondo, Y. & Adachi, K. Identification by single-particle soot photometer of black carbon particles attached to other particles: Laboratory experiments and ground observations in Tokyo. *Journal of Geophysical Research-Atmospheres* **119**, 1031–1043 (2014).
6. Knox, A. *et al.* Mass Absorption Cross-Section of Ambient Black Carbon Aerosol in Relation to Chemical Age. *Aerosol Science and Technology* **43**, 522–532 (2009).
7. Cappa, C. D. *et al.* Radiative Absorption Enhancements Due to the Mixing State of Atmospheric Black Carbon. *Science* **337**, 1078–1081 (2012).
8. Lack, D. A. *et al.* Brown carbon and internal mixing in biomass burning particles. *Proceedings of the National Academy of Sciences of the United States of America* **109**, 14802–14807 (2012).
9. Liu, S. *et al.* Enhanced light absorption by mixed source black and brown carbon particles in UK winter. *Nat Commun* **6**, (2015).
10. Ueda, S. *et al.* Light absorption and morphological properties of soot-containing aerosols observed at an East Asian outflow site, Noto Peninsula, Japan. *Atmos. Chem. Phys.* **16**, 2525–2541 (2016).
11. Ma, Y. *et al.* Mixing state and light absorption enhancement of black carbon aerosols in summertime Nanjing, China. *Atmospheric Environment* **222**, 117141 (2020).
12. Cappa, C. D. *et al.* Light Absorption by Ambient Black and Brown Carbon and its Dependence on Black Carbon Coating State for Two California, USA, Cities in Winter and Summer. *J. Geophys. Res. Atmos.* **124**, 1550–1577 (2019).
